# Telerehabilitation, A Viable Option in Patients with Persistent Post-COVID Syndrome: A Systematic Review

**DOI:** 10.3390/healthcare11020187

**Published:** 2023-01-07

**Authors:** María Ángeles Valverde-Martínez, Remedios López-Liria, Jesús Martínez-Cal, María Jesús Benzo-Iglesias, Lucía Torres-Álamo, Patricia Rocamora-Pérez

**Affiliations:** Health Research Centre, Department of Nursing, Physiotherapy and Medicine, University of Almería, Carretera del Sacramento s/n, La Cañada de San Urbano, 04120 Almería, Spain

**Keywords:** home care, long COVID, physiotherapy, post-COVID-19, telerehabilitation, virtual

## Abstract

The number of patients with post-COVID-19 syndrome continues to increase considerably, having serious healthcare, social and economic repercussions. The objective of this study is to describe the effectiveness of telerehabilitation to alleviate the symptoms of post-COVID-19 syndrome. A systematic review was conducted using the information available on four databases (PubMed, Medline, Scielo and PEDRo) on these patients until November 2022. The MeSH search terms were: Post-COVID syndrome, Post-COVID-19, Long COVID, Telerehabilitation, Physiotherapy, Rehabilitation, Virtual, Home care. Six articles were included which provided information on 140 patients, detailing their symptomatology, assessment, treatment and monitoring. The variables measured were dyspnea, fatigue, physical performance and quality of life. All studies included aerobic and anaerobic exercises. Most notable among the techniques used were rib cage expansion exercises, respiratory control and thoracic cage stretching, patient education, Mindfulness and virtual reality games to address physical, mental and relaxation aspects. The use of telerehabilitation could be an effective tool for the treatment of persistent symptoms after suffering from COVID-19. It has been shown in these studies that patients improve both their physical performance and their quality of life.

## 1. Introduction

There have been 13,441,941 confirmed cases of COVID-19 in Spain, of which Omicron was the dominant variant [1,2]. However, it is estimated that the actual number of positive cases is much higher [3]. Between approximately 10–20% of COVID-19 patients experience persistent symptoms following the illness, which can last for weeks or months [4].

The post-COVID syndrome, also called persistent or prolonged COVID or post-acute COVID syndrome, is defined as the collection of signs and symptoms that develop during or after a compatible infection with COVID-19, which last more than 3 months and can be explained by no alternative diagnosis [5,6]. Underlying respiratory or psychiatric issues, the female sex, presenting more than five symptoms in the acute phase of the infection and the presence of comorbidities were found to be among the risk factors [7,8]. Understanding risk factors is a research priority at the UK National Institute for Health and Care Excellence for managing their long-term effects [9].

It is a systemic disorder with various and changeable clinical symptoms (relapse-remission), encompassing chronic ailments such as fatigue and dyspnea (most commonly), post-exertional malaise, headache, throat and ear pain, abdominal, joint and/or muscle pain, skin rashes, nausea, diarrhea, chest pain, palpitations, cognitive disturbance, sustained tachycardia, orthostatic intolerance and/or neurological disorders such as, notably, postural orthostatic tachycardia syndrome (POTS) and dysautonomia in addition to greater probability of suffering stress, depression, irritability, insomnia, confusion or frustration [5,6,8,9,10]. Post-COVID-19 syndrome can manifest independently of the acuteness of the initial infection, hospitalization status or age [7]. The physio-pathological mechanism of the illness it still not clearly understood [11]. Despite being a multisystemic illness with long-term implications, to date there is no specific treatment with which to manage these patients [6]. The disability associated with post-COVID-19 syndrome appears to have a considerable impact on healthcare services, as treating these patients implies both an overwhelming demand for care and a high economic cost [12,13,14]. For these reasons, attention must be focused on managing symptoms and physical and mental rehabilitation [6].

As a result of the pandemic, telerehabilitation—remote medicine applied to the field of rehabilitation—became a firmly established practice in our society via the use of information and communication technologies (ICT) which provide remote services, such as assessment, prevention, treatment and education [15,16,17]. Among its advantages we find greater participation and co-responsibility on the part of the patient, personalization of the therapeutic plan with a rigorous follow-up of the prescribed treatment and, in general, good perception by patients of the service provided, reducing waiting times and favoring accessibility [16,18,19]. However, a number of negative aspects have been observed, including technological barriers and users’ lack of knowledge and of ICT skills [19].

Technological advances and Internet availability afford the opportunity to develop suitable protocols for telerehabilitation through videoconferences, phone calls, mobile applications and other resources [20]. Several modalities of telerehabilitation exist which are implemented worldwide [21,22]: synchronous—patient and therapist connected via a device in real time; asynchronous—remote monitoring of the adapted intervention, but without the presence of a therapist during the session; or a combination of both. Currently, telerehabilitation is used for the treatment of chronic illnesses (cardiological, cerebrovascular, multiple sclerosis and arthritis), proving to be just as effective as conventional rehabilitation [20,23,24].

The number of patients with post-COVID-19 syndrome continues to increase considerably due to the fact that the SARS-CoV2 virus in various forms (mutations) continues to circulate around the world (and within countries) unchecked despite mass vaccination campaigns and having serious repercussions, socially (the physical and mental health of infected individuals), economically (treatment costs, sick leave) and at the healthcare level (inundated services) [13,24]. There has been one review addressing telerehabilitation for patients with COVID-19 [25], but none have focused specifically on the effectiveness of these interventions among patients with persistent post-COVID-19 syndrome.

The objective of this systematic review is to describe the effectiveness of telerehabilitation to alleviate the symptoms of post-COVID-19 syndrome.

## 2. Materials and Methods

In November 2022, a systematic review was carried out following the PRISMA Statement guidelines [26,27]. It was registered in the PROSPERO database—the international prospective register of systematic reviews (CRD42022372274).

A search was conducted among articles published in PubMed, Medline, Scielo and PEDRo using the following MeSH terms: “Post-COVID syndrome”, “Telerehabilitation”, “Long COVID”, “Physiotherapy”, “Rehabilitation”, “Post-COVID-19”, “Virtual”, “Home care”. In addition, manual searches were carried out for those references found in other related articles that were considered to be of potential interest.

The PICOS eligibility criteria (Participants, Intervention, Comparison, Outcomes and Study design) were used for article selection. The participants would be individuals diagnosed with persistent post-COVID-19 syndrome. The intervention would consist of applying a rehabilitation and/or physiotherapy treatment remotely or virtually, compared to a different modality of rehabilitation and/or physiotherapy treatment or none at all. The results were measured using variables such as aerobic and anaerobic performance, fatigue, depression and quality of life. As for the study type, the only articles chosen would be those featuring clinical trials, quasi-experimental studies or cohort studies.

The inclusion criteria included articles published between 2020 and 2022 on persistent post-COVID-19 syndrome that described the telerehabilitation treatment applied. The exclusion criteria included articles with patients testing positive for COVID-19 or other associated pathologies, or clinical guides, systematic reviews and/or meta-analyses.

The search strategy is described in Table 1.

Study selection, data extraction and management.

Having applied the search strategy, two researchers (R.L.-L. and M.Á.V.-M.) selected and analyzed a total of 178 articles by title and abstract. Following this initial filter of the studies considered potentially relevant, 20 articles in their entirety were read in detail. When any discrepancies arose between the researchers, a third party was consulted (P.R.-P.).

**Figure 1:**
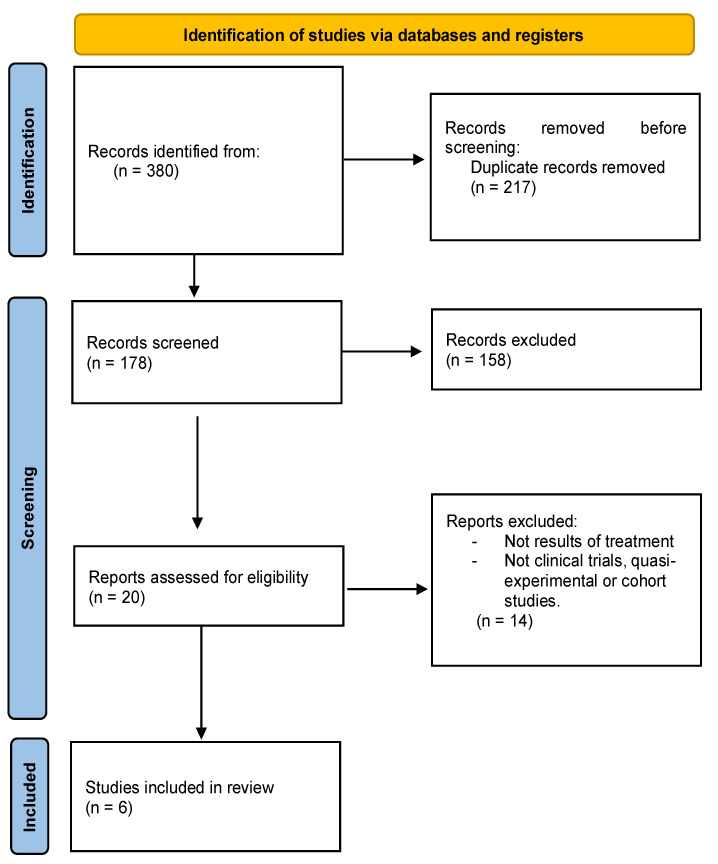
PRISMA flowchart of articles selection process.

Finally, a total of six articles met the proposed objective and criteria for this review (Figure 1).

The evaluation of the methodological quality of the articles included was carried out using the PEDro scale [28], which scores from 0 (poor quality) to a total of 10 points (good quality).

## 3. Results

Six articles were included which provided information on 140 patients with persistent post-COVID-19 syndrome, detailing the symptomatology manifested, the assessment, the telerehabilitation intervention applied and monitoring.

Below, Table 2 displays a summary of the most important information from the selected studies.

In addition, a content analysis was conducted to identify the most important variables in each of the articles.

### 3.1. Participants’ Characteristics

The average age of the participants was 50.11 years old. As for gender, in three studies [13,29,33] most of the participants were women, while another [31] featured a greater proportion of men. The study by Plaza [32] was equally balanced among men and women, while the study by Colas [30] did not specify the sex of the participants at all.

None of the studies had significant demographic differences worth noting.

### 3.2. Main Symptomatology

The main focus of all the studies, except for one which addressed post-COVID-19 patients with a variety of physical and mental symptoms, was the symptomatology related to fatigue and dyspnea. However, the work by Pelhivan [31] also included patients experiencing pain.

### 3.3. Description of Telerehabilitation Procedure and Necessary Resources

Regarding the different telerehabilitation modalities, four of the studies included used live videoconferencing to carry out supervision of the telerehabilitation program [13,30,31,32]. Two others [23,29] propose a program of exercises without supervision by means of a mobile application or virtual reality technology.

All studies include aerobic and anaerobic exercises. Four feature respiratory work with rib cage expansion exercises, respiratory control and thoracic cage stretching [13,31,32,33]. Two emphasize educating the patient as an essential aspect within the rehabilitation program [13,30]. One study covers Mindfulness [32] and another [29] uses virtual reality games to address physical and mental aspects and relaxation.

Only two studies propose a preliminary training for the participant to ensure proper technique and understanding of the exercise program. One proposes a week of rehabilitation (3 one-hour sessions: 45 min aerobic exercises and 15 min anaerobic) at a hospital facility [30] and another [29] proposes that the initial sessions be supervised face to face by a previously trained physiotherapist, followed by written instructions supplied to the patient.

Albeit most studies have no need for tools or materials, the Colas study [30] requires a frontal camera device, a good Internet connection, access to a stationary bike, an exercise mat and a spacious and quiet area where exercises can be conducted. The study by Groenveld [29] supplies participants with a VR headset.

The studies included which use a comparison group [30,31,33] compare telerehabilitation with a traditional rehabilitation program [30], a brochure with similar exercises to those of the telerehabilitation group [31] or simply the provision of educational instructions at the start of the study [33].

The durations of the various interventions were four weeks [30,32], six weeks [29,31,33] and seven weeks [13]. One study does not specify whether there were any adverse effects, [32] and only one study [29] observed dizziness, migraine, blurry vision, altered mood or cervical pain.

All the studies featured both an initial and final assessment, but one study [33] also included a long-term assessment 24 weeks after the intervention, while another [13] conducted follow-up assessments both one and three months after the intervention. Al-though two studies [31,33] do not specify the duration of the session, the time proposed in the articles ranges between 30 min and one hour [13,29,30,32]. The study by Groenveld [29] indicates that 30 min per session is sufficient.

Remote medical monitoring was carried out in all the studies, while psychological and dietary monitoring was conducted on an optional basis [30]. One study monitored by means of VR data and a patient diary, in addition to a 24-h helpline [29].

### 3.4. Variables Measured

At the respiratory level, fatigue is evaluated using the Chalder Fatigue Score (CFS-11) [30]; dyspnea is evaluated with the Modified British Medical Research Council Dyspnea Scale (mMRC Dyspnea Scale) [13,31,33], the Borg Scale [29,30,32], the Modified Borg Dyspnea Scale (MBDS) [13] and the Mahlers Index [32].

With respect to symptomatic status and depression, these are assessed with the Vi-sual Analogue Scale (VAS) and Beck’s Depression Inventory (BDI). Anxiety was evaluated with the State-Trait Anxiety Inventory (STAI) [32].

Physical performance is evaluated in all the articles [13,29,30,31,33], except in the Plaza study [32], by means of the Short Physical Performance Battery (SPPB) [31], the Timed Up and Go Test (TUG) [29,31], the Six-minute walk distance (6 MWD) [33] and the Six-minute walk test (6 MWT) [13,29,30]. One study also evaluates physical function using the Patient-Specific Complaints Questionnaire (PSC) and the Nottingham Extended Activities of Daily Living Scale score (NEADL Scale Score). Anaerobic performance is evaluated with muscular strength tests, such as the grip test [29,30] and the 30-Second Sit to Stand Test (30-CTS) [29].

Quality of life is measured using the 12-Item Short Form Survey (SF-12) [29,33], the Saint George’s Respiratory Questionnaire (SGRQ) [13,31] and the EuroQol-5D [32,33]. One study [29] also includes other measures such as the Hospital Anxiety and Depression Score (HADS), Cognitive Failure Questionnaire (CFQ) and a modified satisfaction questionnaire of the intervention. The study by Colas [30] is the only one that does not evaluate quality of life.

### 3.5. Intervention Effects

All the studies coincide in the effectiveness of their respective telerehabilitation programs, with benefits for physical performance, symptomatology and quality of life, among other aspects. In said programs, the exercises were personally adapted to each patient according to their basal conditions.

One study which evaluated muscular strength indicates that this aspect failed to improve for any group [30] and another investigation states that it improved more in the telerehabilitation group than in the control group [33].

The study by Li [33], despite having a larger number of dropouts (28 participants), displays satisfactory adherence to the telerehabilitation program. The rest of the studies had two [30], three [13], six [31] and seven [29] participants drop out for different reasons, or they simply did not specify any reason [32].

The treatments are useful, safe and low cost, requiring no tools or special equipment to be carried out [13,29,30,31,32]. What is more, one study shows that the effects of telerehabilitation can be maintained for seven months (long term) [33].

### 3.6. Level of Evidence and Quality of Articles Included

The PEDro Scale [28] was used to evaluate the methodological quality of the clinical trials included (results are displayed in Table 3). A clinical trial is considered to have rigorous methodological quality when its score is greater than or equal to 7 points and low quality when it is lower than 6 points out of 10 [28].

The studies analyzed obtained a moderate to rigorous methodological quality [31,33], except one which was of low quality [30].

## 4. Discussion

This systematic review describes recent studies that use telerehabilitation as treatment for patients with persistent symptoms after having suffered from COVID-19, a highly debilitating and frequent ailment in the world today. The results reveal that telerehabilitation could be a useful, suitable and quality resource for treating such patients.

This review observed a mean age of 50 years old among patients, who were predominantly female, which coincides with the review by Cabrera-Martimbianco [34] that relates advanced age and female gender to post-COVID-19 syndrome. It is worth noting the importance of this fact since a recent meta-analysis by Ceban (2022) [3] reveals that a third of patients with post-COVID had persistent symptoms for twelve or more weeks after infection.

The pandemic and the situation generated by COVID-19 have intensified the use of telerehabilitation in the healthcare sector. There is an increasing number of studies that have proven the efficacy of telerehabilitation in other pathologies, although, on occasions, it has been difficult to establish generalizations due to the heterogeneity of the interventions [35].

Also observed among the studies focused on telerehabilitation was an emphasis on evaluating physical performance, without delving into the area of mental health, despite the dramatic repercussions the pandemic has had on physical, psychological and social wellbeing [36,37,38]. However, standard protocols following a case of COVID-19 recommend complete multidisciplinary rehabilitation, in which it is essential to assess and work on psychosocial aspects by means of therapeutic education [38]. Three of the studies included in this review [13,30,31] address this point to improve the emotional components of therapy. Furthermore, intervention protocols were found that support this idea, such as that of Carpallo-Porcar [39]. The study by Harenwall [40] proposes a 6-week VR program with a biopsychosocial approach, encouraging the participant to actively address their own symptomatology, providing them with knowledge and abilities through exercise, self-control, an action plan and problem-solving skills, all managed by an interdisciplinary team (psychologist, physiotherapist, occupational therapist, dietitian, speech and language therapist and a personal support navigator).

Protocols such as that of Turan (2022) [41] compare telerehabilitation under remote supervision with exercise at home, while Lei [42] opts for a pulmonary telerehabilitation program. Both works show similar parameters to those included in the present study (physical function, quality of life, health status, dyspnea and strength) [41,42].

The idea that telerehabilitation can serve as a valid and effective resource for the treatment of patients with post-COVID-19 symptoms [13,29,30,31,32,33] is also supported by a recent review by Vierira (2022) [25], whose clinical trials show that telerehabilitation improves physical function and reduces dyspnea among such patients. Another study [24] conducted on patients with chronic obstructive pulmonary diseases, chronic respiratory disease or COVID-19, suggests that pulmonary telerehabilitation is not inferior to conventional rehabilitation programs and chooses a hybrid model to maintain face-to-face contact at specific moments (e.g., at the initial assessment or when exercises are prescribed). In contrast, most of the studies included in this review conduct both the initial assessment and the prescription of exercises remotely.

Other studies support following a telerehabilitation program over the course of an average of 30 days to improve dyspnea, muscular fatigue and tolerance to exercise [43]. Said time frame is also supported by this review, as the intervention of the studies included were at least a month in duration.

With regard to the use of VR, the study by Wen [24] shows that, when compared to other traditional methods, VR proves interesting and is used successfully for cognitive rehabilitation. However, the study by Groenveld [29] with VR showed no effects on cognitive function. Harenwall [40], in contrast, indicates that VR improves quality of life among patients. Most of the studies on telerehabilitation which evaluated quality of life report significant improvements following treatment [13,29,31,32,33].

Our review highlights respiratory symptomatology, primarily dyspnea and fatigue, as the most prevalent for post-COVID-19 syndrome [22,29,30,31,32,33], a finding supported by other studies also revealing this symptomatology [44,45]. Among the proposed treatments are respiratory physiotherapy, which plays a key role in increasing endurance, decreasing dyspnea and fatigue, as well as improving functionality and quality of life [46,47,48]. Numerous studies opt for respiratory physiotherapy protocols to be conducted remotely [21,49,50].

Telerehabilitation not only proves beneficial among patients with post-COVID-19 syndrome, but also for various other ailments, such as degenerative osteoarthritis, lumbago, multiple sclerosis, heart disease and other respiratory illnesses [21]. However, it is essential to incorporate ICTs into clinical resources and for healthcare professionals to be able to identify patients that are suitable candidates for receiving telerehabilitation (such as patients who do not respond well to other therapies, have financial limitations, little available time, children in their care, or those in need of self-control strategies, education and/or advice on carrying out exercises, etc.) [51]. On the other hand, if we pay attention to the variable dropout rate of the included studies, we see that for the study of Li et al. [33] that this was 28/120 (23%), whilst for the study of Groenveld et al. [29] it was 5/47 with a significant number of patients also experiencing dizziness. This kind of data carries implications with respect to which patients have the most to gain from telerehabilitation, compared to those who do not. In recent years, the scientific literature has stated that telehealth should not replace in-person or face-to-face care; instead, it is an additional tool at the disposal of healthcare professionals to better adapt to the patient’s circumstances if it indeed offers more advantages and benefits [18]. However, an adequate evaluation of patients is necessary before entering telerehabilitation programs, as well as having personalized programs for specific subgroups of patients with long-term COVID and including the participation of multidisciplinary caregivers in these programs.

The limitations of this review are related to the scant sample of studies included, whereas larger samples would generate more evident and reliable results that could be extrapolated. Admittedly, it is difficult to establish a comparison of results since the sparse works available to date set varied goals and thus target outcomes are also different. Furthermore, the long-term complications caused by COVID-19 are still being observed, due to the recent emergence of this pathology and consequent post COVID conditions. Therefore, it is expected that in the future there will be more evidence in the scientific literature regarding the assessment of treatments effectiveness, which will serve as a guide in the therapeutic management of these conditions. Moreover, the use of telerehabilitation in the healthcare field continues to be low, even though the pandemic favored the expansion of this modality. Future investigations are necessary in order to identify the barriers of telerehabilitation for professionals and patients so that it can be better implemented. Telerehabilitation plays an important role in public health emergencies, while allowing greater continuity of care with more flexibility. There is an increased scientific and research interest in telerehabilitation worldwide, including resource-limited countries. However, there are still limitations to its application in relation to the lack of a legislative framework, data privacy, patient safety, reimbursement and/or health coverage as well as established international guidelines, all these factors limiting its implementation in care practice. New lines of research are needed to address useful forms of telerehabilitation with legal and political guidelines that respect the rights of individuals [52].

With regard to practical application, this work intends to be a tool that promotes the use of telerehabilitation if its application is beneficial to reducing pressure within the healthcare system generated by the repercussions of the pandemic in our society and if it obtains similar results to conventional therapies.

Research challenges and opportunities for Telerehabilitation.

Many COVID-19 survivors worldwide suffer from persistent symptoms, impairment of function and reduced quality of life [13,33]. Experts agree on the need for an individualized program with a multimodal approach, not only aiming at restoring physical functioning, but also offering cognitive rehabilitation when needed [29]. An exercise program, starting early and adapted to the symptoms with a focus on returning to daily activities, reduces fatigue, risk of frailty, sarcopenia, cognitive loss and depressive symptoms in individuals with long COVID-19 [30,31].

Telerehabilitation exercise programs and therapeutic patient education are proving to be a good alternative treatment method with less need for technical rehabilitation equipment for long COVID-19 patients, improving their quality of life and symptomatic status in the home environment [13,30,31,33]. Telerehabilitation allows patients a great deal of autonomy in choosing exercises in both the physical and mental domains, taking into consideration the real needs and wishes of the patients with this condition [13,29]. Serving as an example for the implementation of low-budget and tool-free exercise at home, telerehabilitation programmes are suitable for large-scale implementation dependent on smartphone coverage, digital literacy and the availability of therapists for remote supervision and consultations [13,33]. A wide range of virtual reality applications motivates patients to exercise, improving therapy adherence and self-efficacy [29]; adherence to the telerehabilitation is satisfactory and no serious adverse events occur [33].Telerehabilitation with an integrative and person-centred approach is reported to be feasible and well accepted by patients in comparison with standard rehabilitation, although sometimes technology is perceived as difficult to use or patients are simply not familiar with smartphone technology [30,31,33]. Older patients may be slower when using the applications than younger patients, because they are not used to navigating through some application menus. Age differences in acceptation, usability, tolerance and the effects of VR have been described in numerous papers [29]. A potential factor affecting dizziness and nausea, both symptoms of “virtual reality sickness”, is a prolonged time per session (being recommend not to exceed 30 min) and possible latency in the software of physical exercising applications [29].

In our systematic review, we identified a small number of studies with post-COVID-19 telerehabilitation methods, although multidisciplinary rehabilitation teams should perform cardiopulmonary and musculoskeletal programs, neurorehabilitation and psychological rehabilitation to treat COVID-19 sequelae patients [32]. There is a need for guidelines and protocols to reduce the variability of physiotherapy approaches, such as different types of physical and performance assessment, different indications for self-administered exercises at home and different follow-up schemes. Furthermore, longer term follow-up would be necessary in order to assess if the benefits of this telerehabilitation persist over time. The results of this review have provided substantive and organizational leads to future research regarding health, social and economic impact of telerehabilitation use in post–COVID condition.

## 5. Conclusions

The studies included in this review state that the use of telerehabilitation could be an effective tool for the treatment of persistent symptoms following cases of COVID-19. It has been shown to improve both patients’ physical performance and their quality of life in most of these studies. Certainly, telerehabilitation seems to be useful, but there needs to be some caveats placed around how it is used in patients with long COVID-19, because a subgroup of patients presented adverse effects, such as dizziness.

More research is required to establish effective treatment protocols adapted to patients with post-COVID-19 syndrome which facilitate quality random clinical trials that allow researchers to determine the efficacy of telerehabilitation in comparison with conventional treatments or in a combination of both.

## Figures and Tables

**Table 1:** Databases and Search Terms.

Databases and Search Terms	Results	Selected Articles
PUBMEDo “Post-COVID syndrome” AND “Telerehabilitation” “Long COVID” AND “Telerehabilitation” “Coronavirus” AND “Telerehabilitation” “Post-COVID-19” AND “Telerehabilitation” “Telerehabilitation” AND “Physiotherapy” AND “Post-COVID-19” “Rehabilitation” AND “virtual” AND “Post-COVID-19” “Post-COVID syndrome” AND “Telerehabilitation” AND “Home care”	307	5
SCIELO, MEDLINE “Post-COVID syndrome” AND “Telerehabilitation” “Long COVID” AND “Telerehabilitation” “Coronavirus” AND “Telerehabilitation” “Post-COVID-19” AND “Telerehabilitation” “Telerehabilitation” AND “Physiotherapy” AND “Post-COVID-19” “Rehabilitation” AND “virtual” AND “Post-COVID-19” “Post-COVID syndrome” AND “Telerehabilitation” AND “Home care”	62	0
PEDRo “Post-COVID syndrome” AND “Telerehabilitation” “Long COVID” AND “Telerehabilitation” “Coronavirus” AND “Telerehabilitation” “Post-COVID-19” AND “Telerehabilitation” “Telerehabilitation” AND “Physiotherapy” AND “Post-COVID-19” “Rehabilitation” AND “virtual” AND “Post-COVID-19” “Post-COVID syndrome” AND “Telerehabilitation” AND “Home care”	11	1

**Table 2:** Summary of key information from the articles.

Author and Year	Type of Study Participants and Intervention Symptomatology	Monitoring and Duration	Variables	Results
Groenveld et al. [29] 2022	Cohort study Patients with post-COVID condition N = 47 5 dropouts. Mean age: 54 years old 22 men and 32 women - VR exercises at home via games. Physical, cognitive and relaxation exercises. Initial sessions supervised in person by a physiotherapist. Symptoms: variety of physical and mental symptoms.	No supervision. Weekly semi-structured telephone call. 24/7 helpline Monitoring through patient comments and daily VR data. Initial and final assessment. 30-min session, at least once a week; 6 weeks.	Acceptability: intervention satisfaction questionnaire (+2 questions), Mentality Test. Use, tolerance and safety: daily. Physical performance: 6 MWT, TUG, grip test, 30-CST, PSC, NEADL. Fatigue: Borg scale. Mental function and quality of life: HADS, SF-12, Positive Health Questionnaire, CFQ.	Conducted 3 to 4.5 times/week for 94–115 min/week. Significant improvements in 6 MWT (*p* < 0.001), grip test (*p* = 0.01), 30-CST (*p* = 0.02), Borg scale (*p* = 0.03) and PSC (*p* < 0.001). No significant changes in HADS (*p* = 0.08). No decrease in CFQ (*p* = 0.11). Increased scores in Positive Health Questionnaire (*p* = 0.04) and SF-12, at physical (*p* = 0.049) and mental (*p* = 0.01) level. The use of VR for self-managed physical and mental exercise at home is viable and safe. 33 patients reported adverse effects (dizziness, headache).
Calvo-Paniagua et al. [13] 2022	Quasi-experimental, prospective, multicenter study, of only one group PCR patients—with fatigue and dyspnea. N = 71 3 dropouts Mean age: 48.5 years old 26 men and 42 women Exercise program with telehealth method: - Health education - Respiratory work - Physical conditioning Symptoms: fatigue and dyspnea	Videoconference (Zoom) Initial and final assessment, at 1 month and 3 months 18 sessions, 40 min 3 times/week (alternate days) 7 weeks	Physical performance: 6-MWT. Dyspnea: MBDS and mMRC Quality of life: SGRQ	Improvements in physical strength (*p* < 0.001), dyspnea (*p* < 0.001) and quality of life (*p* < 0.001). Changes maintained 3 months afterwards. Useful way of managing and monitoring a large number of patients. Significant reduction in economic burden of managing these patients.
Colas et al. [30] 2022	Nonrandomized clinical trial Patients with post-COVID-19 fatigue N = 17 patients 2 dropouts Mean age: 52.1 years old GC (n = 6): 12 sessions traditional rehab GI (n = 9): 12 supervised sessions of APA: 3 sessions at hospital, 9 at home. No adverse effects Symptoms: fatigue	Videoconference. Smartwatch. Weekly remote medical monitoring. Optional: psychological and/or dietary monitoring. Initial and final assessment. 3 sessions/week. 4 weeks	Physical performance: Aerobic: 6-MWT. Anaerobic: manual grip test (dynamometer) Fatigue: CFS-11 and Borg scale. FEV-1: Spirometry.	Significant effect in 6-MWT (*p* = 0.019) in both groups Reduced fatigue in both groups (*p* = 0.010) Neither group improved their anaerobic capacities (*p* = 0.400) Good alternative to a face-to-face program for patients with persistent symptoms.
Pelhivan et al. [31] 2022	Randomized clinical trial. Patients with symptoms 4 weeks after COVID-19 N = 40 6 dropouts Mean age: 47 years old 25 men and 9 women CG (n = 16): 1 workout session and a brochure with exercises similar to IG. IG (n = 17): - Patient education - Steady run/ walk. - Respiratory exercises. - Mobility exercises and standing squats. No adverse effects. Symptoms: fatigue and symptomatic status (pain)	Videoconference in real-time with supervision. Monitoring by pneumologist with optimum medication. 3 sessions/week 6 weeks	Dyspnea: mMRC scale Symptomatic status: EVA for fatigue and pain Physical performance: TUG and SPPB Quality of life: SGRQ and BDI	The telerehabilitation program improved symptoms and quality of life and increased physical performance. There was a statistically significant difference between the groups in SGRQ activity (*p* = 0.035) and total (*p* = 0.042) scores. More symptomatic improvement was found in the telerehabilitation group. Allows the implementation of an effective, useful and safe program, at a low cost and without special equipment.
Plaza et al. [32] 2022	Quasi-experimental study Patients with post-COVID syndrome N = 20 No dropouts Mean age: 48.5 years old 10 men and 10 women Exercise program using Google Forms. - Respiratory physiotherapy. - Jacobson relaxation - Mindfulness No adverse effects mentioned Symptoms: dyspnea	Remote supervision by a physiotherapist through a digital platform. 10 sessions 3 times/week 45 min/session Before and after intervention	Quality of life: EuroQol-5D Dyspnea: Mahlers Index, Borg scale Anxiety: STAI	Significant decrease in dyspnea (*p* < 0.001) and state and trait anxiety, *p* = 0.004 and *p* = 0.001, respectively. Significant increase in quality of life (*p* = 0.016) Appears to be an effective treatment approach for post-COVID patients.
Li et al. [33] 2022	Randomized clinical trial. Patients with post-COVID, with persistent dyspnea N = 120 28 dropouts Mean age: 50.61 years old 53 men and 66 women CG (n = 55; 5 excluded): Brief informative instructions at beginning of study IG (n = 36; 23 excluded) Exercise program (mobile application) - Respiration control and thoracic expansion. - Strength exercises and lower limbs. Without adverse effects. Symptoms: Dyspnea (score 2–3 mMRC)	No supervision of exercises. Monitoring via teleconsultation (once/week) Initial and final housecall visit and 24 weeks after intervention.	Physical performance: 6-MWT/6-MWD and muscular strength of lower limbs. Pulmonary function: spirometry QLRH: SF-12 (post-treatment and after 28 weeks) Fatigue: mMRC scale (at 2 and 4 weeks, post-treatment and at 28 weeks)	The parameters of pulmonary function improved in both groups. 6-MWT average improved by 80.2 m in the IG. Treatment effect *p* < 0.001. Muscular strength of lower limbs improved to a greater extent in IG *p* < 0.001 after treatment. In the physical component, SF-12 increased to a greater extent in the IG *p* = 0.004 following treatment and *p* = 0.045 in the monitoring. Thus, the telerehabilitation program was superior in terms of functional capacity, muscular strength and physical QLRH. Satisfactory adherence. It is economical and suitable for large-scale implementation.

N = number of patients; CG = control group; IG = intervention group; rhb = rehabilitation; VR = virtual reality; min = minutes; 6 MWT=: 6-Minute Walk Test; TUG = Timed Up and Go Test; 30-CST = 30 Seconds Sit to Stand Test; PSC = Patient-Specific Complaints Questionnaire; NEADL: Nottingham Extended Activities of Daily Living Scale score; HADS = Hospital Anxiety and Depression Score; SF12 = Short Form-12; CFQ = Cognitive Failure Questionnaire; PCR = polymerase chain reaction; MBDS = Modified Borg Dyspnea Scale; mMRC=: Modified British Medical Research Council; CFS-11 = Chalder Fatigue Score; FEV-1 = forced expiratory volume in 1 s; VO2 = maximal oxygen uptake; EVA = Visual analog scale; SPPB = Short physical performance battery; SGRQ = Saint George’s Respiratory Questionnaire; BDI = Beck Depression Inventory; EuroQol-5D = quality of life score; STAI = State-Trait Anxiety Inventory; 6 MWD = Six-minute walk distance; QLRH = quality of life related to health.

**Table 3:** Evaluation of the methodological quality of the articles included with the PEDro Scale [28].

	PEDro Scale Items	Specified Selection Criteria	Random Assignment	Hidden Assignment	Similar Groups	Blinded Subjects	Blinded Therapists	Evaluators Blinded	Adequate Follow-Up	Intention to Treat Analysis	Results of Comparisons between Groups	Point Measures of Variability	Total Score
RCTs													
Colas et al. [30], 2022		X	N	N	X	N	N	N	X	X	X	X	6
Pehlivan et al. [31], 2022		X	X	X	X	X	X	N	N	X	X	X	9
Li et al. [33], 2021		X	X	X	X	X	N	X	N	X	X	X	9

N: the criterion is not satisfied; X: the criterion is satisfied.

## Data Availability

No new data were created.
